# Efficacy of Ciprofloxacin for Treatment of Cholera Associated with Diminished Susceptibility to Ciprofloxacin to *Vibrio cholerae* O1

**DOI:** 10.1371/journal.pone.0134921

**Published:** 2015-08-13

**Authors:** Wasif Ali Khan, Debasish Saha, Sabeena Ahmed, Mohammed Abdus Salam, Michael Louis Bennish

**Affiliations:** 1 icddr, b: International Centre for Diarrhoeal Disease Research, Dhaka, Bangladesh; 2 Centre for International Health, Department of Preventive and Social Medicine, School of Medicine, University of Otago, Dunedin, New Zealand; 3 Mpilonhle, Mtubatuba, South Africa; Beijing Institute of Microbiology and Epidemiology, CHINA

## Abstract

**Objective:**

We identified a poor clinical response to treatment of cholera with a single 1 g dose of ciprofloxacin, a standard treatment for cholera.

**Methods:**

To determine reasons for the poor response and better therapeutic approaches we examined the minimal inhibitor concentration (MIC, n = 275) and disc-diffusion zone sizes (n = 205) for ciprofloxacin and nalidixic acid of *V*. *cholerae* O1 strains isolated in Bangladesh from 1994 to 2012, and reexamined data from 161patients infected with *Vibrio cholerae* O1 recruited in four clinical trials who received single- or multiple-dose ciprofloxacin **for** treatment of cholera and compared their clinical response to the *V*. *cholerae* O1 susceptibility.

**Results:**

Although all 275 isolates of *V*. *cholerae* O1 remained susceptible to ciprofloxacin using standard MIC and disc-diffusion thresholds, the MIC^90^ to ciprofloxacin increased from 0.010 in 1994 to 0.475 μgm/ml in 2012. Isolates became frankly resistant to nalidixic with the MIC^90^ increasing from 21 μgm/ml in 1994 to >256 μgm/ml and 166 of 205 isolates from 1994 to 2005 being frankly resistant using disc-diffusion testing. Isolates resistant to nalidixic acid by disc-diffusion testing had a median ciprofloxacin MIC of 0.190 μgm/ml (10^th^-90^th^ centiles 0.022 to 0.380); nalidixic acid-susceptible isolates had a median ciprofloxacin MIC of 0.002 (0.002 to 0.012).The rate of clinical success with single-dose ciprofloxacin treatment for nalidixic acid-susceptible strains was 94% (61 of 65 patients) and bacteriologic success 97% (63/65) compared to 18% (12/67) and 8% (5/67) respectively with nalidixic acid-resistant strains (P<0.001 for both comparisons). Multiple-dose treatment with ciprofloxacin had 86% and 100% clinical and bacteriologic success rates respectively in patients infected with nalidixic acid-susceptible strains of *V*. *cholerae* O1 compared to clinical success 67% and bacteriologic success 60% with nalidixic acid-resistant strains.

**Conclusions:**

Single-dose ciprofloxacin is not effective for treating cholera caused by *V*. *cholerae* O1 with diminished susceptibility to ciprofloxacin, and nalidixic acid disc-diffusion testing effectively screens for such isolates.

## Introduction

Antimicrobial agents reduce the duration and volume of diarrhea by approximately half in severe cholera, and are thus an important adjunct to fluid therapy in the management of this illness.[1, 2] A variety of agents have been used effectively in the treatment of cholera–including tetracyclines, chloramphenicol, the nitrofuran agent furazolidone, ampicillin, and trimethoprim-cotrimoxazole.[3] *Vibrio cholerae* O1 and O139 –the causative agents of cholera–have developed resistance to all of these agents, however.[4]

This resistance prompted–beginning in the 1990s –the increasing use of fluoroquinolones in the treatment of cholera.[5] The fluoroquinolones are attractive agents for use in cholera because of their very good activity in-vitro, high concentrations in the gut lumen (the site of infection), their high therapeutic ratio, and their relatively long half-life.[5] These characteristics led to the fluoroquinolones successfully being used as single-dose-therapy, [6, 7] or as a single daily dose,[8] for the treatment of cholera. The use of short courses of therapy is an important consideration given the logistic difficulties in administering multi-dose-therapy in the impoverished settings where cholera is endemic, especially during the recurrent epidemics that characterize cholera.

Although *V*. *cholerae* O1 isolates from infections in Bangladesh remain susceptible to fluoroquinolones when using standard threshold criteria for determining resistance, more recent controlled trials–in which the fluoroquinolone agent was used as the comparator drug–found diminished efficacy of the fluoroquinolone agent (in this case ciprofloxacin) in achieving a clinical or bacteriologic cure.[9]

Ciprofloxacin resistance in *Vibrio cholerae* associated with efflux pump and target gene mutation has already been reported in the Indian subcontinent. [10] Recent study suggests that the Haitian variant Cholera Toxin-producing *Vibrio cholerae* O1 El Tor strains with reduced susceptibility to ciprofloxacin are spreading to other regions of India. [11] In addition occurrence of cholera incidence in Zimbabwe by *V*. *cholerae* strains with reduced susceptibility against ciprofloxacin was also reported [12]

In this study we explore the reasons for this diminished response, describe methods useful in poor developing country settings for identifying strains of *V*. *cholerae* O1 with a diminished response to quinolone therapy, as well as identifying alternative therapeutic approaches. To do this we reexamined data from four clinical trials of antimicrobial agents in the treatment of cholera conducted between 1992–2005. We determined changes in MIC of ciprofloxacin against *V*. *cholerae* O1 during this period, the relation between the ciprofloxacin MIC and clinical and bacteriologic response to therapy, and the utility of nalidixic acid as a screening test for strains of *V*. *cholerae* O1 with diminished susceptibility to ciprofloxacin.

## Methods

### Ethics Statement

Data analyzed in this study were from previously approved protocols by the Ethical Review Committee (ERC) of the icddr, b, where the same investigators were involved. Thus there was no further requirement of signed informed consent however, ERC was informed where we maintained patient records / information anonymous and de-identified prior to analysis.

### Clinical Information

The clinical information used in this study comes from 161 patients who were infected with *V*. *cholerae* O1 and was enrolled in four randomized controlled trials conducted between 1992 and 2005 of the treatment of cholera, in which either single or multiple-dose ciprofloxacin was one of the treatment arms. Table 1 of the manuscript describes the number of adult patients from each of 4 clinical trials that were included in the analysis. [9, 13–15] All of the patients were adult, treated with either single (n = 132) or multiple-dose (n = 29) ciprofloxacin and completed a 5 day period stay at the hospital. Definitions of clinical and bacteriologic cure are detailed in those reports, but briefly they are cessation of watery stools within 48-hours of the initiation of antimicrobial therapy, and the inability to isolate *V*. *cholerae* after 48 hours of administration of study medication, respectively.

**Table 1:** Randomized controlled trials from which 161 adult patients infected with *V*. *cholerae* O1;treated with ciprofloxacin and completed 5day study were included in this analysis.

Study author(reference)	Years patients enrolled	Number of patients in clinical response analysis: total number of patients in the study	Ciprofloxacin dose administered
Khan[13]	1992	15:75 (20%)	500 mg 12h for 3d
Khan[14]	1993–1995	66:272(24%)	1 g single dose
Saha[9]	2002–2004	65:198 (33%)	1 g single dose
Salam[15]	2005	15: 35 (43%)	500 mg 12h for 3d

There were 580 patients in total in these four studies, of whom 161 (28%) were infected with *V*.*cholerae* O1; treated with ciprofloxacin and completed 5 day study

### Collection of *V*. *cholerae* Isolates and Susceptibility Testing

Susceptibility testing by MIC was conducted on 275 *V*. *cholerae* O1 isolates obtained from patients with clinical cholera from 1994 to 2012. Two hundred five (75%) of these isolates also had susceptibility to ciprofloxacin and nalidixic acid determined using the disc diffusion method.

Among the 275 *V*. *cholerae* O1 strains, 121(44%) were enrolled in clinical studies of drug efficacy in treating cholera. However only 75 of these 121 strains, which were isolated in the year between 2003 to 2005 were included in clinical response analysis. Rest 46 out of 121 strains were isolated between the period 2001 to 2005 from patients of other clinical trials who were not included in clinical response analysis either due to not being an adult or not receiving ciprofloxacin for treatment. The remaining 154 (56%) *V*. *cholerae* O1 strains were obtained from clinical microbiology laboratory who were also from patients admitted to the Dhaka Hospital of icddr, b.

V. *cholerae* O1 were isolated and identified by standard microbiological techniques.[16] Antimicrobial susceptibility was determined by the disc-diffusion method using nalidixic acid (30μg) and ciprofloxacin (5μg) disks according to methods described in National Committee for Clinical Laboratory Standards (NCCLS) currently known as Clinical Laboratory Standard Institute (CLSI);[17] and by MIC using the E-test (bioMérieux SAMarcy l'Etoile, France, previously ABBIODISK, Solna, Sweden) according to manufacturer’s instruction. The CLSI recommended threshold levels for determining nalidixic acid susceptibility against *V*. *cholerae* using the disc-diffusion technique and MIC values are: susceptible, ≥ 19 mm of growth inhibition using the disc-diffusion method, or ≤ 16 μg/ml on MIC; intermediate, 17–31 μg/ml on MIC (there is no cutoff value for NA for the disc-diffusion method; and resistant), <19 mm of growth inhibition on disc-diffusion, or ≥32μg/ml on MIC. For ciprofloxacin measures of susceptibility against *Enterobacteriaceae* (there are no specific recommendations for *V*. *cholerae*) the respective disc diffusion and MIC threshold values are: susceptible- ≥ 21 mm or ≤ 1 μg/ml; intermediate—16–20 mm or 2 μg/ml; and resistant- ≤ 15 mm or ≥ 4 μg/ml respectively.[18] Quality control strain Escherichia coli ATCC 25922 and/or Staphylococcus aureus ATCC 25913 were included in each run of susceptibility testing.

### Statistical methods

The χ^2^ test with continuity correction was used to determine the significance of differences in proportions between groups, and Fisher’s exact test was done if the predicted size of any cell was five or less. The Mann-Whitney U test was used to assess the significance of differences in continuous variables. The binomial method was used to calculate differences in medians between groups, and the confidence intervals for those differences. The Newcombe method was used to determine confidence intervals for differences in proportions. All tests of significance were two-tailed. Analysis was conducted using IBM SPSS Statistics for Windows v17 (IBM, Armonk, New York).

## Results

### Susceptibility

All 275 *V*. *cholerae* O1 isolates collected during this 19-year period were susceptible to ciprofloxacin by MIC and disc-diffusion testing using standard threshold criteria (Table 2). The MIC^50^ and MIC^90^ for ciprofloxacin, while remaining in the susceptible range, increased dramatically during this period. The MIC^50^ for ciprofloxacin increased from 0.002 μg/ml in 1994 to 0.250 μg/ml in 2003 (a 125-fold increase) and the MIC^90^ during the same period from 0.010 μg/ml to 0.250 μg/ml (a 25-fold increase). There were much less dramatic changes in disc-diffusion testing results. The 90^th^ centile for the disc-diffusion zone size to ciprofloxacin decreased only from 27 to 21 mm.

**Table 2:** Nalidixic acid and ciprofloxacin MIC^50^and MIC^90^ of 275 isolates of *V cholerae* O1 by year and source of strains obtained.

Year	Number of strains tested	Nalidixic acid		Ciprofloxacin		Source
		MIC^50^	MIC^90^	MIC^50^	MIC^90^	
1994	13	0.50	21	0.002	0.010	CLS
1995	23	0.70	102	0.002	0.016	CLS
1996	9	8	>256	0.012	0.023	CLS
1997	9	16	>256	0.023	0.032	CLS
1998	12	16	>256	0.023	0.029	CLS
1999	8	8	64	0.023	0.250	CLS
2000	10	16	88	0.032	0.230	CLS
2001	29	>256	> 256	0.032	0.125	CT
2002	10	>256	>256	0.032	0.244	CT
2003	41	>256	>256	0.250	0.250	CT
2004	22	>256	>256	0.250	0.341	CT
2005	19	>256	>256	0.380	0.750	CT
2006	10	>256	>256	0.250	0.250	CLS
2007	10	>256	>256	0.250	0.380	CLS
2008	10	>256	>256	0.380	0.380	CLS
2009	10	>256	>256	0.380	0.488	CLS
2010	10	>256	>256	0.250	0.488	CLS
2011	10	>256	>256	0.250	0.250	CLS
2012	10	>256	>256	0.250	0.475	CLS

Values are (μg/ml)

CT–Clinical trial; CLS–Clinical Laboratory Services, icddr,b

During these 19 years *V*. *cholerae* O1 isolates became frankly resistant to nalidixic acid. The MIC^50^ for nalidixic acid increased from 0.5 μg/ml to >256 μg/ml (>512 fold) and the MIC^90^ from 21 μg/ml to >256 μg/ml (>12 fold). The 90^th^ centile for disc-diffusion zone size to nalidixic acid decreased from 16 mm to 7 mm. The disc-diffusion and MIC testing showed 70% concordance in identifying resistant strains; 145 isolates out of 205 *V cholerae* O1 strains determined to be nalidixic acid-resistant by disc-diffusion testing were also resistant by MIC testing. Isolates resistant by disc-diffusion to nalidixic acid (n = 167) had a median ciprofloxacin MIC of 0.190μg/ml (10^th^-90^th^ centiles 0.022 to 0.380) compared to 0.002 μg/ml for nalidixic acid susceptible (n = 38) isolates (10^th^-90^th^ centiles 0.002 to 0.012).

### Clinical response

Of the 161 adult patients infected with *V*. *cholerae* O1 in these trials who received ciprofloxacin 132 (82%) received single-dose (SD) ciprofloxacin (1 g orally) and 29 (18%) received multiple-dose (MD) therapy with ciprofloxacin (either 500 mg orally every 12 hours for 3 days) (Table 1). In 65 of 132 patients receiving SD ciprofloxacin, infection was caused by nalidixic acid-susceptible strains whereas rest 67 patients were infected with nalidixic acid-resistant strains. Again in 14 of 29 patients receiving MD therapy with ciprofloxacin, infection was caused by nalidixic acid-susceptible strains and rest 15 patients were infected with nalidixic acid-resistant strains.

The 79 patients infected with nalidixic acid-susceptible strains of *V*. *cholerae* O1 were older than the 82 patients infected with nalidixic acid-resistant strains, and had more severe disease (Table 3). They had a longer duration if illness, had a greater volume of stool in a 4 hour observation period before the initiation of antimicrobial therapy, and required more intravenous fluids. Ciprofloxacin treatment, however, was dramatically more effective in patients infected with nalidixic acid-susceptible strains of *V*. *cholerae*. The rate of clinical success was 95%, compared to 27% in those infected with nalidixic acid-resistant isolates (P <0.001) and the rate of bacteriologic success was 97% versus 17% (P <0.001). The group with infection resistant to nalidixic acid also fared worse on all secondary measures of disease outcome–diarrhea duration, volume of stool, and volume of fluids required (Table 3).

**Table 3:** Admission characteristics and response to ciprofloxacin therapy in 161 patients infected with nalidixic acid-susceptible and nalidixic acid-resistant strains of *V*. *cholerae* O1.

Variable	Nalidixic acid-susceptible* (n = 79)	Nalidixic acid-resistant (n = 82)	Difference (95% CI)	P
**Before drug administration**				
Age (yrs)	30 (22, 40)	25 (20, 30)	5 (1 to 8)	0.004
Duration (h)	12 (7, 16)	7 (6, 12)	3 (1 to 4)	0.001
Number of stool since onset of illness	12 (7, 25)	10 (6, 16)	2 (0 to 4)	0.095
Stool output (ml/kg/h) during 4-hour observation period after rehydration	14 (9, 19)	12 (8, 14)	3 (1 to 5)	0.007
Intravenous infusion (ml/kg/h) during four-hour observation period	8 (0, 18)	0 (0, 0)	6 (2 to 9)	< 0.001
**Primary outcomes**				
Clinical success (n, %)	73(92%)	22 (27%)	0.66(0.52to 0.75)	< 0.001
Bacteriological success (n, %)	77 (97%)	14 (17%)	0.80 (0.69 to 0.87)	< 0.001
**Secondary outcomes**				
Diarrhoea duration (h)	30 (24, 42)	75 (42, 92)	-42 (-48 to –36)	< 0.001
Patients vomited during study (n, %)	32 (41%)	51 (62%)	-0.22 (-0.36 to –0.06)	0.007
Patients with IV fluid reinstitution (n, %)	45 (57%)	40 (49%)	0.08 (-0.07 to 0.23)	0.345
Fluid balance after start of study drug (ml/kg) ^‡^				
Watery stool	148 (98, 257)	313 (129 to 488)	-135 (-200 to –71)	< 0.001
Vomiting	0 (0, 22)	30 (0, 64)	-17 (-31 to 0)	< 0.001
Intravenous fluids	22 (0, 139)	71 (0, 878)	0 (-197 to 0)	0.034
Oral rehydration solutions	195 (145, 265)	1137 (548, 1736)	-938 (-1201 to –736)	< 0.001

Values are median (25^th^, 75^th^ centiles) unless noted

* Based on disc-diffusion method

^‡^ Based on discharge weight

For patients with nalidixic-acid susceptible *V*. *cholerae* infections single and multiple dose ciprofloxacin therapy were both highly effective–with clinical rates of cure of 94% and 86% respectively (Table 4). Single-dose ciprofloxacin therapy was, however, significantly inferior in treating patients with nalidixic-acid resistant *V*. *cholerae* infection (Table 5). Clinical success was achieved in only 18% of patients with nalidixic acid-resistant *V*. *cholerae* O1 infections treated with a single dose, compared to 67% in those who received three-day therapy (P < 0.001) (Table 5). Rates of bacteriology success were 8% and 60% respectively (P < .0001) (Table 5).

**Table 4:** Clinical and bacteriologic response to single-dose or multiple-dose ciprofloxacin therapy in patients infected with nalidixic acid-susceptible strains of *V*. *cholerae* O1.

Variable	Single-dose (n = 65)	Multiple-dose (n = 14)	Difference (95% CI)	P
**Primary outcomes**				
Clinical success (n, %)	61 (94)	12 (86%)	-0.081 (-0.053 to 0.341)	0.287
Bacteriological success (n, %)	63 (97)	14 (100)	-0.03 (-0.11 to 0.19)	1.0
**Secondary outcomes**				
Diarrhoea duration (h)	30 (24, 39)	24 (24, 48)	0 (-10 to 6)	0.551
Patients vomited during study (n, %)	29 (45)	3 (21)	0.23 (-0.05 to 0.42)	0.140
Patients with IV fluid reinstitution (n, %)	40 (62)	5 (36)	-0.26 (-0.02 to 0.48)	0.135
**Fluid balance after start of study drug (ml/kg)** *				
Watery stool	151 (101, 257)	124 (82, 251)	18 (-41 to 80)	0.472
Vomiting	0 (0, 23)	0 (0, 1)	0 (0 to 12)	0.088
Intravenous fluids	45 (0, 141)	0 (0, 76)	2 (0 to 68)	0.093
Oral rehydration solutions	186 (136, 240)	240 (203, 336)	-64 (-118 to -13)	0.019

Values are median (25^th^–75^th^ centiles) unless noted

* Based on discharge weight

**Table 5:** Clinical and bacteriologic response to single-dose or multiple-dose ciprofloxacin therapy in patients infected with nalidixic acid-resistant strains of *V*. *cholerae* O1.

Variable	Single-dose(n = 67)	Multiple-dose(n = 15)	Difference(95% CI)	P
**Primary outcomes**				
Clinical success (n, %)	12 (18)	10 (67)	-0.49 (-0.68 to -0.22)	< 0.001
Bacteriological success (n, %)	5 (8)	9 (60)	-0.53 (-0.73 to -0.27)	< 0.001
**Secondary outcomes**				
Diarrhoea duration (h)	78 (60, 96)	42 (30, 72)	30 (12 to 48)	0.001
Patients vomited during study (n, %)	45(67)	6 (40)	0.27 (0.001 to 0.50)	0.076
Patients with IV fluid reinstitution (n, %)	34 (51)	6 (40)	0.11 (-0.16 to 0.34)	0.571
**Fluid balance after start of study drug (ml/kg)** *				
Watery stool	328 (169, 505)	183 (69, 315)	127 (12 to 248)	0.031
Vomiting	36(0, 84)	0 (0, 40)	17 (0 to 44)	0.037
Intravenous fluids	250 (0, 1029)	0 (0, 93)	199 (0 to 743)	0.027
Oral rehydration solutions	1398 (905, 1880)	241 (146, 404)	1119 (771 to 1410)	< 0.001

Values are median (25^th^–75^th^ centile) unless noted.

* Based on weight discharge

## Discussion

There are at least four important findings from this study. The first is that *V*. *cholerae* O1 is becoming less susceptible to ciprofloxacin in Bangladesh, the country with the highest burden of cholera infection. The second is that current thresholds for determining antimicrobial susceptibility of *V*. *cholerae* to ciprofloxacin in-vitro are not predictive of clinical response in cholera, and that strains with diminished susceptibility have a poorer clinical response to therapy. The third is that determining susceptibility to nalidixic acid using the disc diffusion method is a good screening tool for identifying *V*. *cholerae* O1 strains with diminished susceptibility to ciprofloxacin. Fourth, the clinical and bacteriologic response to single-dose ciprofloxacin therapy may differ from response to multi-dose therapy depending on the level of susceptibility of the infecting strain of *V*. *cholerae*.

During the 19 years covered in this study the MIC^90^ of *V*. *cholerae* strains to ciprofloxacin increased 45 fold—from 0.010 μg/ml in 1994 to 0.475 μg/ml in 2012. This pattern of decreased susceptibility to fluoroquinolones, but not frank resistance, is one that has been seen with a number of enteric pathogens, especially Salmonella,[19–21] but also in Shigella [21, 22], Campylobacter,[23] and also previously in *Vibrio cholerae* O1.[24–26] The decreased resistance to the fluoroquinolones is almost invariably associated with frank resistance to nalidixic acid, and usually results from a single mutation in the *gyrA* gene coding the enzyme–DNA gyrase–that is the target for the quinolones.[27] Additional mutations–either in *gyrA* or in other genes encoding fluoroquinolone targets- is required for frank resistance to the fluoroquinolones (based on Clinical Laboratories and Standards Institute recommended interpretive criteria) to occur.

As has been reported for Salmonella and *Neisseria gonorrhea* infections, [28–31] the clinical response to ciprofloxacin therapy in patients infected with strains of *V*. *cholerae* O1 resistant to nalidixic acid and with diminished susceptibility to ciprofloxacin was sub-optimal. This problem appears to be worse with short-course therapy.[32] In this study the risk of clinical failure of ciprofloxacin treatment for those patients infected with a nalidixic acid-resistant strain of *V*. *cholerae* O1 was almost 15 times that of those infected with a nalidixic acid-susceptible strain – 73% versus 5%.

Predicting clinical response to therapy based on in-vitro determinations of drug activity remains an inexact science. The usual pharmacokinetic and pharmacodynamic measures used are the peak serum drug concentration as a multiple of the MIC of the infecting organism, and the ratios of 24-hour area under the serum concentration-versus-time curve (AUC) to MIC. For Salmonella infections with strains with diminished susceptibility to fluoroquinolones, the peak serum concentration in relation to the MIC are less than the desired ratio of 10, and the AUC to MIC ratios are also sub-optimal.[33] That is because peak serum concentrations of ciprofloxacin are usually 2–3 μg/ml, insufficient for strains with ciprofloxacin MIC values of 0.5 μg/ml or greater, as is often the case with nalidixic acid-resistant isolates of Salmonella.

Those pharmacokinetic measures in serum seem appropriate for an infection, such as Salmonella, which is invasive and for which the serum concentrations are likely to be the critical determinant. But gut concentration of drug is thought to be the critical determinant of drug efficacy for an enteric non-invasive organism such as *V*. *cholerae*, for which a number of non-absorbable antimicrobials have proven to be effective.[34] With single-dose therapy peak stool concentrations of ciprofloxacin in cholera patients are 21.0 μg/ml [7]–considerably higher, even for strains with diminished susceptibility—than the optimal 10x the MIC^90^ concentration thought to be required for a clinical response.[33]

The problem with single-dose treatment lies with sustaining those high levels of drug. At 24 hours after single dose therapy peak stool concentrations of ciprofloxacin in cholera patients were only 3.3 μg/ml.[7]This drug concentration is likely to be sufficient for killing *V*. *cholerae* strains with MICs of 0.010 to 0.125 μg/ml that were found in Bangladesh from 1994–2001 (more than 300 and 26 x the MIC^90^ respectively, but less than optimal in 2012 for strains of *V*. *cholerae* (MIC^90^ 0.475 μg/ml) with reduced susceptibility to ciprofloxacin, where the 24hr and 48hr peak stool drug concentration to MIC ratio is 9 and 1 respectively (less than 10).

Nalidixic acid disc-diffusion testing proved a reliable measure for identifying those *V*. *cholerae* O1 strains with decreased susceptibility to ciprofloxacin. 167 (81%) of the 205 strains resistant to nalidixic acid by disc-diffusion had decreased susceptibility to ciprofloxacin, with the MIC^50^ and MIC^90^ being 0.190 μg/ml and 0.380μg/ml respectively. Of the 38 isolates that tested susceptible to nalidixic acid by disc diffusion testing, the MIC^50^ and MIC^90^to ciprofloxacin were 0.002 μg/ml and 0.012μg/ml respectively.

For most clinical laboratories in developing countries, disc diffusion testing is the simplest and least expensive measure of resistance. Tube dilution or automated MIC testing is for the most part not available, and E-tests for determining MICs, although adaptable to developing country settings and used for determining *V*. *cholerae* susceptibility to erythromycin,[35] are considerably more expensive (disc diffusion technique cost is $0.73 compared to E-test cost $7.31). Using resistance to nalidixic acid on disc-diffusion testing as a screening test for identifying Salmonella and *N*. *gonorrhea* isolates with diminished susceptibility to fluoroquinolones has already been recommended.[28, 33]

Although the Clinical and Laboratory Standards Institute (the organization most commonly looked to for standards for susceptibility testing) has published interpretive standards specific for testing of *V*. *cholerae* susceptibility to ampicillin, chloramphenicol, tetracycline and trimethoprim-sulfamethoxazole,[36] no standards specific for testing of nalidixic acid and the fluoroquinolones have been established. Instead, the recommended breakpoints for *Enterobacteriaceae* are commonly used for interpreting *V*. *cholerae* susceptibility.[37] The current recommended threshold values also do not correlate with clinical response to ciprofloxacin in the treatment of *V*. *cholerae*. All 205 *V*. *cholerae* isolates in this study that were tested for ciprofloxacin susceptibility using disc diffusion testing, had zone size ≥ 21 mm, indicating all were susceptible. Thus, there is a need to develop recommendations for interpreting *V*. *cholerae* susceptibility to fluoroquinolones, as has been suggested for Salmonella and *N*. *gonorrhea*.[28, 33] We have already found that recommended interpretive breakpoints do not accurately correlate with clinical outcome for doxycycline treatment of cholera.[7]

Retrospective analyses combining data from a number of studies, such as we have done in comparing the response to single- or multiple dose antimicrobial therapy in patients infected with *V*. *cholerae* O1 with diminished susceptibility to ciprofloxacin, are subject to limitations. The hypothesis is post-hoc, populations can differ, and outcome measures can change. The studies included in this report, though having been conducted over 16 years, benefit from having had the same team conduct them, with the same outcome measures, and in the same location. Most importantly, the magnitude of the effect found was substantial–therapy failed irrespective of single or multi-dose of ciprofloxacin [(60 (73%) out of 82 compared to 4 (5%) out of 79 patients] 15 times more frequently in those infected with a nalidixic acid resistant *V*. *cholerae* infection, and for those infected with a nalidixic-acid resistant isolate, the risk of failure was two time greater if they received single-dose rather than multiple-dose therapy.

In many areas ciprofloxacin is one of the few remaining agents effective against *V cholerae*–recent *V*. *cholerae* strains in Bangladesh have been resistant to virtually every other drug known to be clinically effective in treating cholera.[38] Because of such widespread resistance to older agents ciprofloxacin–most often in a single-dose-has become a drug of choice for treating cholera.[38] Prudence would suggest, however, that recommendations to use single-dose fluoroquinolone therapy be modified to recommend its use only if there is evidence from bacteriologic surveillance that *V*. *cholerae* strains with diminished susceptibility are not commonly present in the community. If such strains are present, an alternative drug known to be active in-vitro and clinically effective in-vivo should be used. Absent such an option multi-dose ciprofloxacin should be used. The efficacy of the latter for treating strains with diminished susceptibility needs to be more fully evaluated in prospective trials.
